# Soluble CD40 Ligand Stimulates the Pro-Angiogenic Function of Peripheral Blood Angiogenic Outgrowth Cells *via* Increased Release of Matrix Metalloproteinase-9

**DOI:** 10.1371/journal.pone.0084289

**Published:** 2013-12-16

**Authors:** Lara Bou Khzam, Rahma Boulahya, Haissam Abou-Saleh, Ahmed Hachem, Younes Zaid, Yahye Merhi

**Affiliations:** 1 Laboratory of Thrombosis and Hemostasis, Montreal Heart Institute, Montréal, Québec, Canada; 2 Qatar Cardiovascular Research Center, Qatar Foundation-Education City, Doha, Qatar; 3 Université de Montréal, Department of Medicine, Montréal, Québec, Canada; Medical University Innsbruck, Austria

## Abstract

The role of endothelial progenitor cells in vascular repair is related to their incorporation at sites of vascular lesions, differentiation into endothelial cells, and release of various angiogenic factors specifically by a subset of early outgrowth endothelial progenitor cells (EOCs). It has been shown that patients suffering from cardiovascular disease exhibit increased levels of circulating and soluble CD40 ligand (sCD40L), which may influence the function of EOCs. We have previously shown that the inflammatory receptor CD40 is expressed on EOCs and its ligation with sCD40L impairs the anti-platelet function of EOCs. In the present study, we aimed at investigating the effect of sCD40L on the function of EOCs in endothelial repair. Human peripheral blood mononuclear cell-derived EOCs express CD40 and its adaptor proteins, the tumor necrosis factor receptor-associated factors; TRAF1, TRAF2 and TRAF3. Stimulation of EOCs with sCD40L increased the expression of TRAF1, binding of TRAF2 to CD40 and phosphorylation of p38 mitogen activated protein kinase (MAPK). In an *in vitro* wound healing assay, stimulation of EOCs with sCD40L increased the release of matrix metalloproteinase 9 (MMP-9) in a concentration-dependent manner and significantly enhanced the angiogenic potential of cultured human umbilical vein endothelial cells (HUVECs). Inhibition of p38 MAPK reversed sCD40L-induced MMP-9 release by EOCs, whereas inhibition of MMP-9 reversed their pro-angiogenic effect on HUVECs. This study reveals the existence of a CD40L/CD40/TRAF axis in EOCs and shows that sCD40L increases the pro-angiogenic function of EOCs on cultured HUVECs by inducing a significant increase in MMP-9 release *via*, at least, the p38 MAPK signaling pathway.

## Introduction

 Endothelial progenitor cells (EPCs) have shown potential value in cell therapy and regenerative medicine [1]. Circulating levels of EPCs have been associated with endothelial damage and the incidence of cardiovascular disease [2-4]. The pioneer study of Asahara et al. demonstrated the potential of circulating CD34^+^ hematopoietic stem cells to differentiate into endothelial cells and induce neovascularization [5]. Recent studies have shown the existence of two subtypes of EPCs; early EPCs, also called cultured angiogenic cells or early outgrowth cells (EOCs), and late EPCs, also named colony-forming EPCs or late outgrowth cells. It has been suggested that EOCs derive from an abundant monocytic cell line [6], whereas late EPCs derive from a rare stem cell source [7,8]. Moreover, it is believed that these two EPC subtypes play different but complementary roles in vascular repair and neovasculogenesis [9-11], where EOCs may not themselves be incorporated at the sites of vascular lesions, as opposed to late EPCs, but they may influence vascular repair in a paracrine manner through the release of various angiogenic factors [6,12,13].

The process of neovascularization relies on the invasive properties of cells through the release of proteolytic enzymes such as matrix metalloproteinases (MMPs), which are required for the degradation of the endothelial basement membrane, thereby favoring cell migration [14]. These proteases regulate the endothelial extracellular matrix turnover by maintaining the physiological balance between pro- and anti-angiogenic factors through the activation of numerous growth factors, such as transforming growth factor beta (TGF-β) and vascular endothelial growth factor (VEGF) [14]. It has been shown that MMPs are also involved in the recruitment and mobilization of progenitor cells from the bone marrow, and favor their incorporation at sites of injury during vascular repair. More specifically, MMP-9 is responsible for the proteolytic cleavage necessary for soluble Kit-ligand (sKitL) release from the bone marrow, which is required for the recruitment of hematopoietic stem cells [14-16]. 

 We have previously shown that the interaction between EPCs and platelets occurs *via* P-selectin, a cell adhesion molecule expressed at the surface of activated platelets. This interaction impairs the function of platelets by inhibiting their activation, aggregation, adhesion and thrombus formation through an increase in COX-2 expression and prostacyclin (PGI_2_) release by EPCs [17]. More recently, we have identified the constitutive expression of CD40 on EOCs [18], a 39-kd glycoprotein member of the tumor necrosis factor receptor family that is expressed on B cells, platelets, macrophages, dendrite cells and endothelial cells [19]. It is well established that activated platelets constitute the main source of its ligand in the circulation, known as soluble CD40 ligand (sCD40L), which is an 18-kd truncated form of the ligand [20-22]. Adaptor proteins, in particular the tumor necrosis factor (TNF) receptor-associated factors (TRAFs), are required to bind to the cytoplasmic tail of CD40 in order to transmit various downstream intracellular signals [19,23].

The CD40L/CD40 axis plays an important role in inflammation mainly by increasing the expression of cell adhesion molecules, pro-inflammatory cytokines, chemokines and MMPs [24-26]. The clinical correlation between significantly elevated levels of CD40L and cardiovascular risk factors has evoked an interest in the implication of the CD40L/CD40 axis in various cardiovascular diseases, such as atherosclerosis and acute coronary syndromes [26-29]. The thrombo-inflammatory processes involved in the induction of endothelial dysfunction play an important role in the development of these diseases [20,25,26], which calls for further investigation into the role of EPCs in endothelial repair. In this regard, it has been shown that sCD40L impairs the function of EOCs by increasing superoxide anion production and decreasing viability and proliferation, leading to accelerated neointimal progression [30]. More recently, we demonstrated that sCD40L reduces the anti-platelet function of EOCs [18]. However, evidence as to whether sCD40L could also influence the angiogenic potential of EOCs is still elusive. This study was therefore designed to determine the effects and the mechanisms of action of sCD40L on the angiogenic function of EOCs in endothelial repair. 

## Materials and Methods

### Ethics statement

This study was carried out according to a protocol approved by the Montreal Heart Institute ethical committee in agreement with the declaration of Helsinki. Informed written consent was obtained from healthy volunteers aged between 20 and 60 years and medication free over 10 days prior to blood collection. 

### Isolation and culture of PBMC-derived EOCs

Culture of EOCs was carried out as previously described [6,31,32]. Briefly, Ficoll-Paque (GE Healthcare) density gradient centrifugation was used to isolate peripheral blood mononuclear cells (PBMCs) from 100 mL of peripheral blood. PBMCs were cultured in complete endothelial growth medium EGM-2 (Lonza Inc.) at 37°C in an atmosphere of 5% CO_2_. To generate EOCs, 1 × 10^6^ PBMCs per cm^2^ were seeded on 6-well fibronectin-coated tissue culture plates (BD Biosciences). Following 4 days of culture, medium was changed to remove non-adherent cells. Additional culturing up to 7 days was allowed to obtain EOCs. Human umbilical vein endothelial cells (HUVECs) were cultured on 0.2% gelatin-coated plates in complete EGM-2 supplemented with 10% FBS.

### Western blot and immunoprecipitation

EOCs and freshly isolated PBMCs were resuspended in PBS, sonicated and mixed with the appropriated volume of 4 × Laemmli loading buffer and heated for 5 min at 95°C. Protein lysates (40 μg) were then separated by SDS-PAGE, transferred onto nitrocellulose membranes (Bio-Rad) and blocked with 5% non-fat milk in TBS-Tween-20 for 1 hour. Membranes were then incubated overnight with primary antibodies (1:1000) against TRAF1, 2, 3, 6 (Cell Signaling), TRAF5 (Lifespan Biosciences) and CD40 (Santa Cruz Biotechnology). Following washing steps, membranes were labeled with horseradish peroxidase-conjugated secondary antibody for 1 hour, washed and bound peroxidase activity was detected by enhanced chemiluminescence (PerkinElmer Life Sciences). The membranes were then stripped to reveal β-actin, as a control for the amount of proteins loaded for each TRAF. Phospho-p38 MAPK (rabbit polyclonal, Thr^180^/Tyr^182^) and total p38 MAPK (Cell Signaling Technology) were also immunoblotted in sCD40L-stimulated EOCs in the presence or absence of 10 μM p38 inhibitor (SB203580, Calbiochem), as described previously [22,33].

For immunoprecipitation, EOCs (1 × 10^6^ cells/mL) were left untreated (baseline) or stimulated for 15 and 30 minutes with 1 μg/mL sCD40L (Alexis Biochemicals), lysed in modified RIPA (EMD Millipore) lysis buffer (1% NP-40, 0.25% deoxycholic acid, 150 mM NaCl, 50 mM Tris-HCl pH 7.4, 1 mM EDTA, 1 mM PMSF, 1 mM sodium-orthovanadate, 1 mM sodium fluoride, 1 μg/mL aprotinin, 1 μg/mL leupeptin, and 2 μg/mL benzamidin) for 1 hour at 4°C, pre-cleared with Protein A agarose beads (EMD Millipore) for 15 minutes at 4°C and incubated overnight with an anti-human CD40 antibody (5 μg/mL) at 4°C. Samples were then incubated with Protein A agarose beads for 1 hour at 4°C, washed with 1 × modified RIPA lysis buffer and heated in 2 × Laemmli buffer for 5 minutes at 95°C. Supernatants were collected and analyzed by Western Blot for TRAF expression [22]. A SDS-PAGE molecular weight standard from Bio-Rad was used to identify the different proteins. All gels were scanned (GS-800, Bio-Rad) and the optical density of each band was quantified using the Quantity One gel analysis program (Bio-Rad) in 50 mm^2^ area for each protein. 

### Gelatin zymography

 EOCs were gently scraped, washed and resuspended in EGM-2 at 4 × 10^6^ cells per mL. The supernatant of untreated (control) and sCD40L-treated (0.1-1 μg/mL) EOCs for 24 hours were collected. Similarly, supernatants were collected from EOCs treated for 24 hours with SB203580 (10 μM). Thereafter, 20 μL samples were mixed with non-reducing Laemmli buffer and loaded on SDS-PAGE gels containing 2 mg/mL gelatin. Gels were washed twice for 15 minutes with 2.5% Triton X-100 washing buffer and incubated overnight in enzyme assay buffer (38 mM Trizma hydrochloride, 13 mM pH 7.5, CaCl_2_ anhydride, 0.05% NaN_3_) in a 37°C bath to allow development of active enzyme bands. Following overnight incubation, gels were washed and stained with 0.2% Coomassie brilliant blue (methanol: acetic acid: water [4:1:6 ratio]) for 1 hour at room temperature. Gels were distained in 40% methanol with 10% acetic acid until gelatinase activity appeared as clear bands against a dark background. Finally, gels were scanned (GS-800, Bio-Rad) and relative band density was quantified using Quantity One (Bio-Rad) in 50 mm^2^ area for each MMP [34].

### Wound scratch healing assay

 EOCs were gently scraped, washed and resuspended in starved EGM-2 (without FBS, VEGF and FGF) at 4 × 10^6^ cells per mL. Untreated (control) or sCD40L-stimulated (1 μg/mL) EOCs, in the presence or absence of 10 μM of a MMP-9 inhibitor (MMP-9 inhibitor I, EMD Millipore), were incubated for 24 hours at 37°C in an atmosphere of 5% CO_2_. HUVECs were cultured on 0.2% gelatin-coated plates until confluence. On the day of the assay, straight line scratches were performed using 100 μL pipette tips. Wells were carefully washed with 1 × PBS to gently remove detached cells, followed by the addition of 150 μL of starved EGM-2 and 150 μL of control or treated EOC supernatants. HUVEC wound healing was observed over an 8-hour period and images were taken at 2 × magnification using an inverted microscope. Quantification of scratches was performed using the T-scratch program (CSE lab ETH, Swiss Federal Institute of Techonology Zurich) by calculating percent wound healing using the wound areas at 0 and 8 hours.

### Statistical analysis

Results are presented as mean ± SEM of at least 3 independent experiments. Statistical comparisons were done using a one-way ANOVA, followed by a Dunnetts-*t*-test for comparison against a single group. Data with p<0.05 were considered statistically significant.

## Results

### EOCs express the inflammatory receptor CD40 and its TRAF adaptor proteins

**Figure 1:**
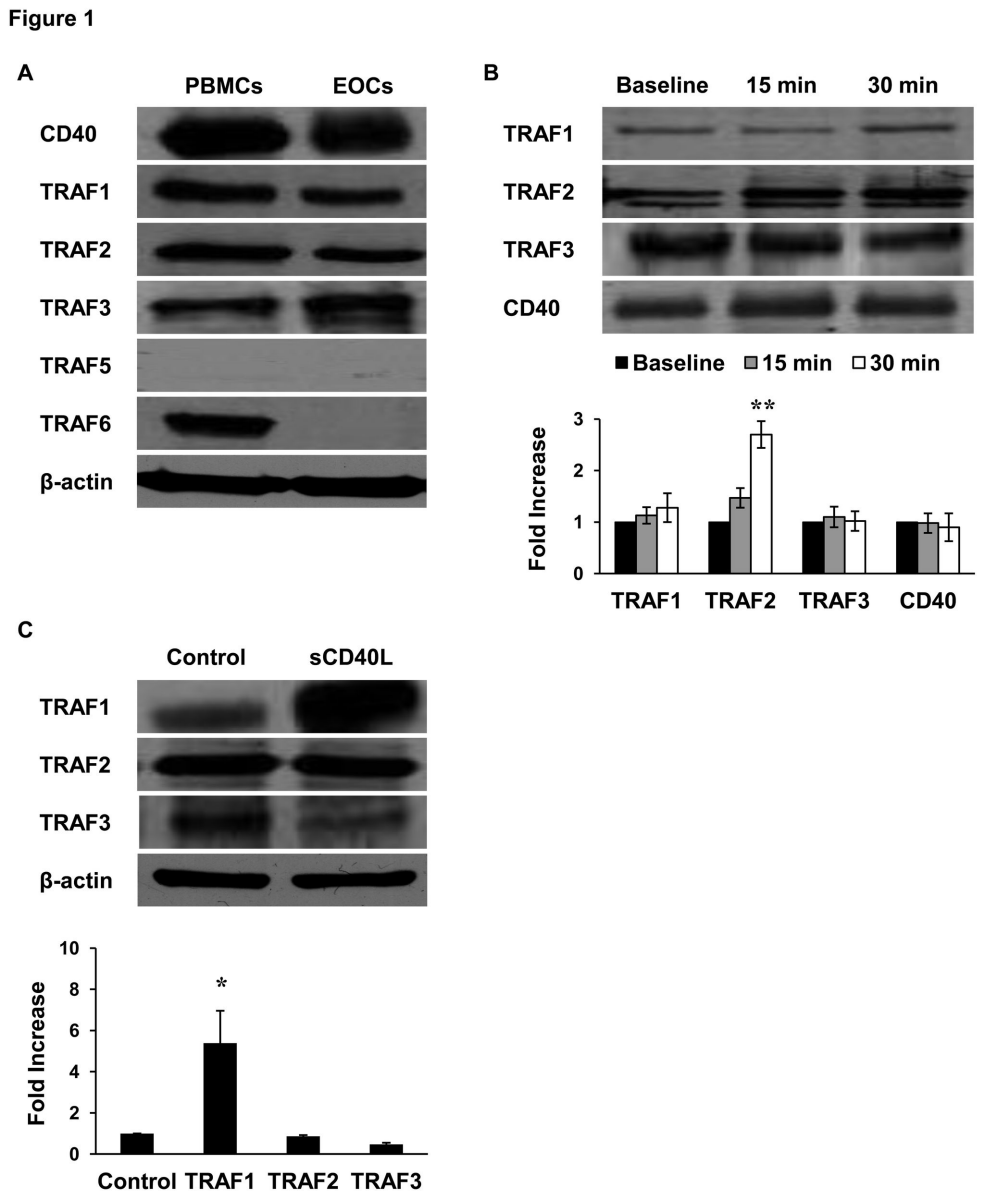
CD40 and TRAF expression and binding to CD40 in EOCs. A) Comparative CD40 and TRAF expression profiles in PBMCs and EOCs, as determined by Western Blots of cell lysates analyzed for CD40, TRAF1, 2, 3, 5 and 6 expressions; the loading control β-actin is representative of the proteins loaded for each TRAF (blots are representative of n ≥ 4). B) Representative blots showing the effect of sCD40L on TRAF association with CD40. EOCs were stimulated with sCD40L (1 μg/mL) for 15 or 30 minutes or left untreated (baseline) and CD40 was immunoprecipitated from lysates using an anti-CD40 monoclonal antibody. Immunoprecipitates were then assayed for TRAF1, 2 and 3 by SDS-PAGE. Histogram represents the mean of data ± SEM of fold increase in optical density over baseline. n ≥ 4, **P<0.01 *vs*. baseline. C) Representative blots showing the effect of sCD40L on TRAF expression in EOCs following 24 hour stimulation with sCD40L (1 μg/mL); the loading control β-actin is representative of the proteins loaded for each TRAF. Histogram represents the mean of data ± SEM of fold increase in optical density over control. n=3, *P<0.05.

Based on recent studies showing the thrombo-inflammatory role of the CD40L/CD40 axis, we first sought to investigate the existence of this axis in EOCs. Figure 1A shows that, like PBMCs, EOCs express CD40. We further analyzed the intracellular signaling components of the CD40L/CD40 axis by identifying the TRAF members present in EOCs. Our results show that EOCs, as well as PBMCs, express TRAF1, 2 and 3, whereas TRAF5 was not detected. However, TRAF6 was expressed in PBMCs and lost during their differentiation into EOCs. These results identify for the first time the TRAF members in EOCs.

### Regulation of TRAF expression and binding to CD40 in response to sCD40L

Having identified the TRAF members expressed in EOCs, we evaluated the effect of sCD40L stimulation on the regulation of TRAF expression and their association with CD40. In immunoprecipitation experiments, we found that TRAF1, 2 and 3 are associated with CD40 at baseline and that only TRAF2 association significantly increased (approximately 3-fold increase) upon 30 minutes stimulation with sCD40L (Figure 1B). Among the TRAFs expressed in EOCs, only TRAF1 was significantly up-regulated (approximately 5-fold increase) in sCD40L-stimulated EOCs (Figure 1C). However, TRAF3 expression was slightly but not significantly decreased.

### sCD40L induces phosphorylation of p38 MAPK in EOCs

**Figure 2:**
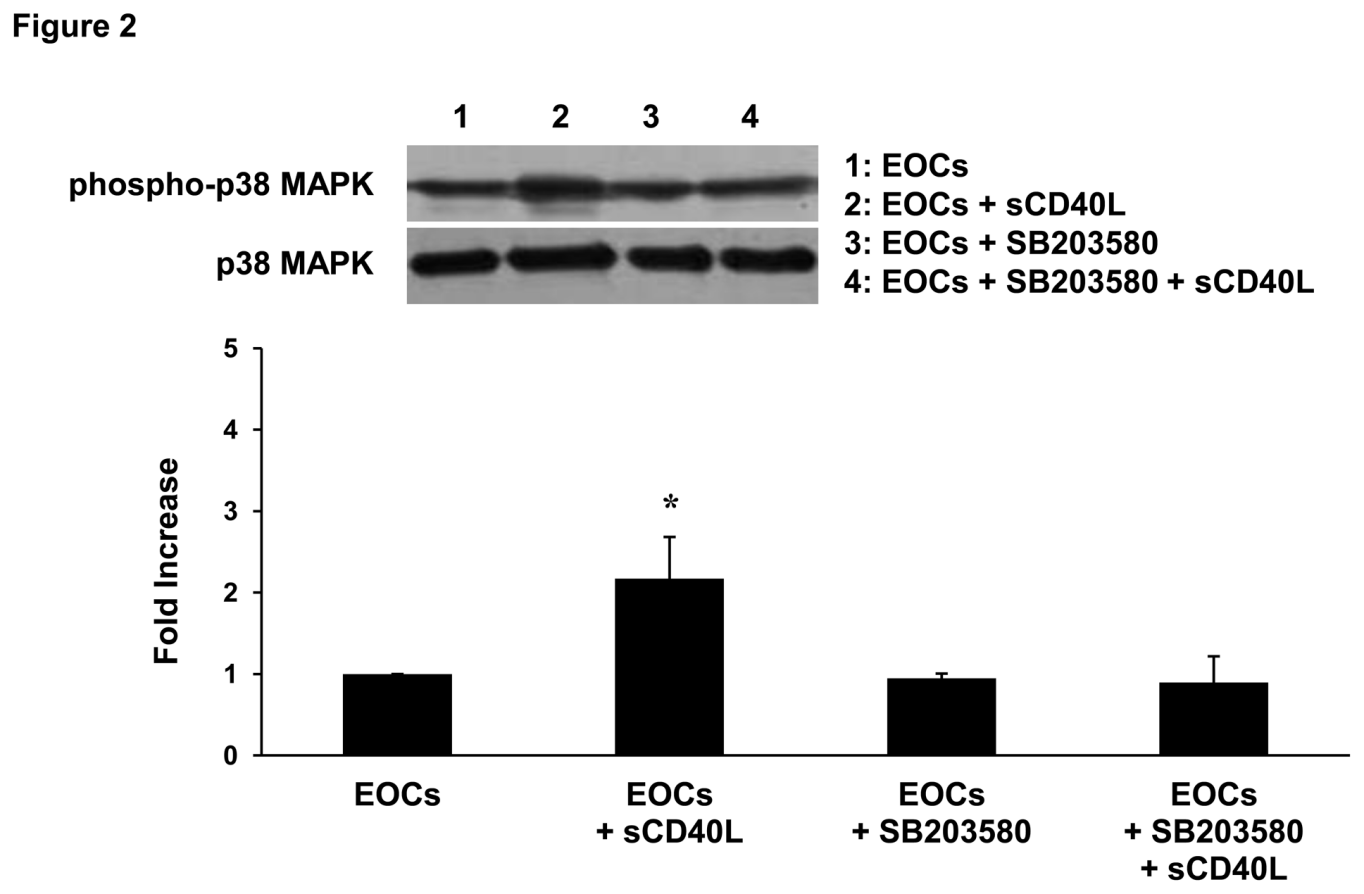
sCD40L induces p38 MAPK phosphorylation in EOCs. Total cell lysates of sCD40L (1 μg/mL)-, p38 inhibitor SB203580 (10 μM)-, and both sCD40L- and SB203580-treated EOCs assessed by SDS-PAGE for phospho-p38 MAPK. The p38 MAPK blot is representative of stripped phospho-p38 MAPK membranes. Histogram represents the mean of data ± SEM of fold increase in optical density over control EOCs. n ≥ 3, *P<0.05 *vs*. other groups.

 Having previously shown the implication of p38 MAPK in platelet function upon sCD40L stimulation [22], we sought to investigate whether this pathway is also involved in EOC function. Interestingly, we have found that sCD40L significantly increased the phosphorylation of p38 MAPK without affecting total p38 MAPK protein levels (Figure 2). Inhibition of p38 MAPK with the specific inhibitor SB203580 reversed the increase in p38 MAPK phosphorylation. These results suggest that sCD40L induces downstream signals in EOCs through at least the p38 MAPK pathway. 

### sCD40L increases MMP-9 release by EOCs *via* p38 MAPK

**Figure 3:**
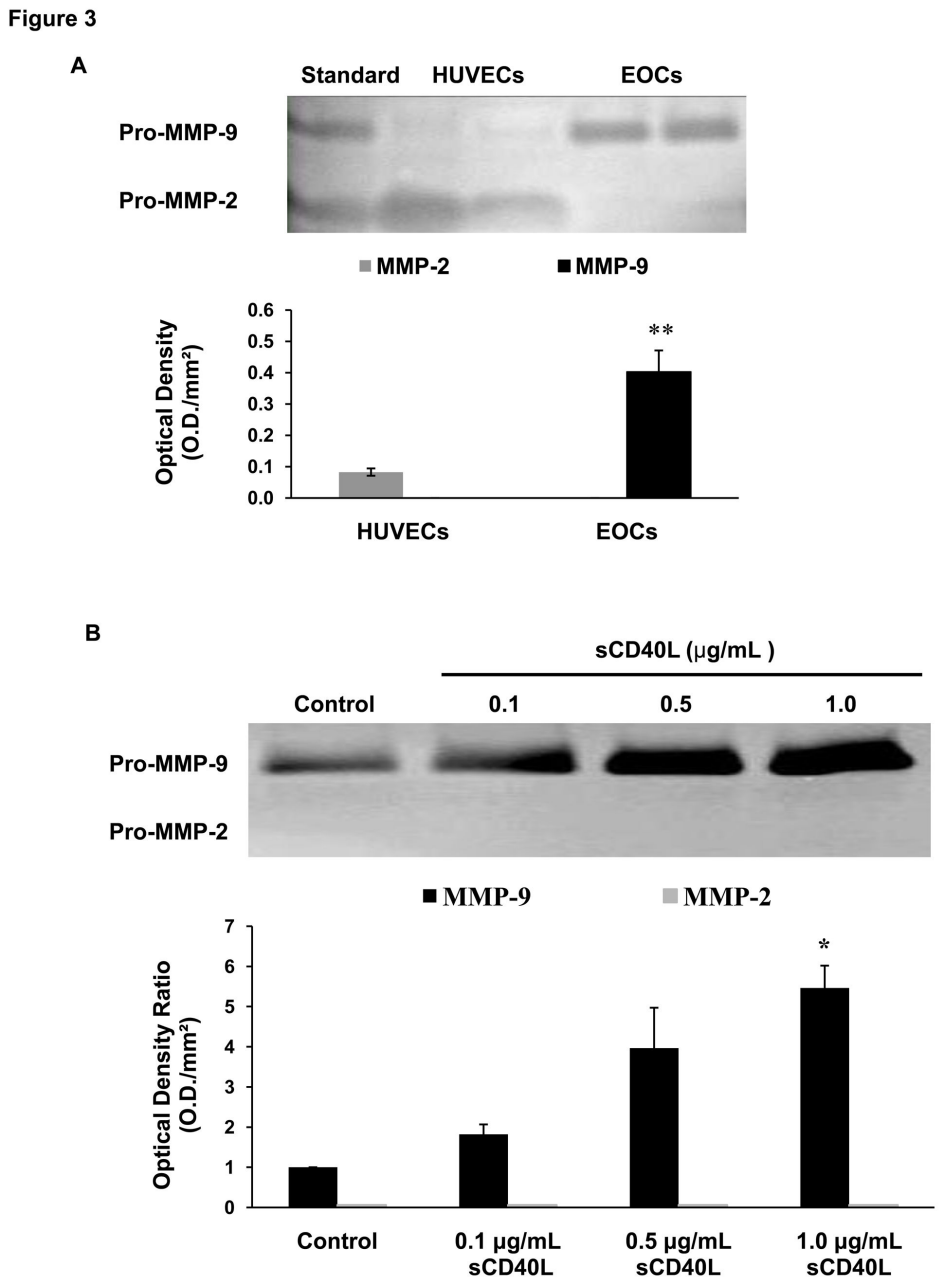
sCD40L increases MMP-9 release by EOCs. A) Representative gelatin zymography gel of MMP-2 and MMP-9 release by HUVECs and EOCs. Histogram represents the mean of data ± SEM of optical density. n ≥ 4, **P<0.01 *vs*. HUVECs. B) Representative gelatin zymography gel showing the release of MMP-9 by control EOCs and the effects of increasing concentration of sCD40L (0.1, 0.5 and 1.0 μg/mL) following 24 hours stimulation of EOCs. Histogram represents the mean of data ± SEM of optical density ratio. n ≥ 3,*P<0.05 *vs*. control.

 In order to characterize the functionality of EOCs, supernatants from EOC cultures were collected and assessed for MMP-2 and MMP-9 activity by gelatin zymography. Interestingly, we found that EOCs release MMP-9 whereas control HUVECs release MMP-2 (Figure 3A). We then investigated the effect of sCD40L on MMP-9 release by EOCs and found that sCD40L induced a significant increase in MMP-9 release in a concentration-dependent manner (Figure 3B). 

**Figure 4:**
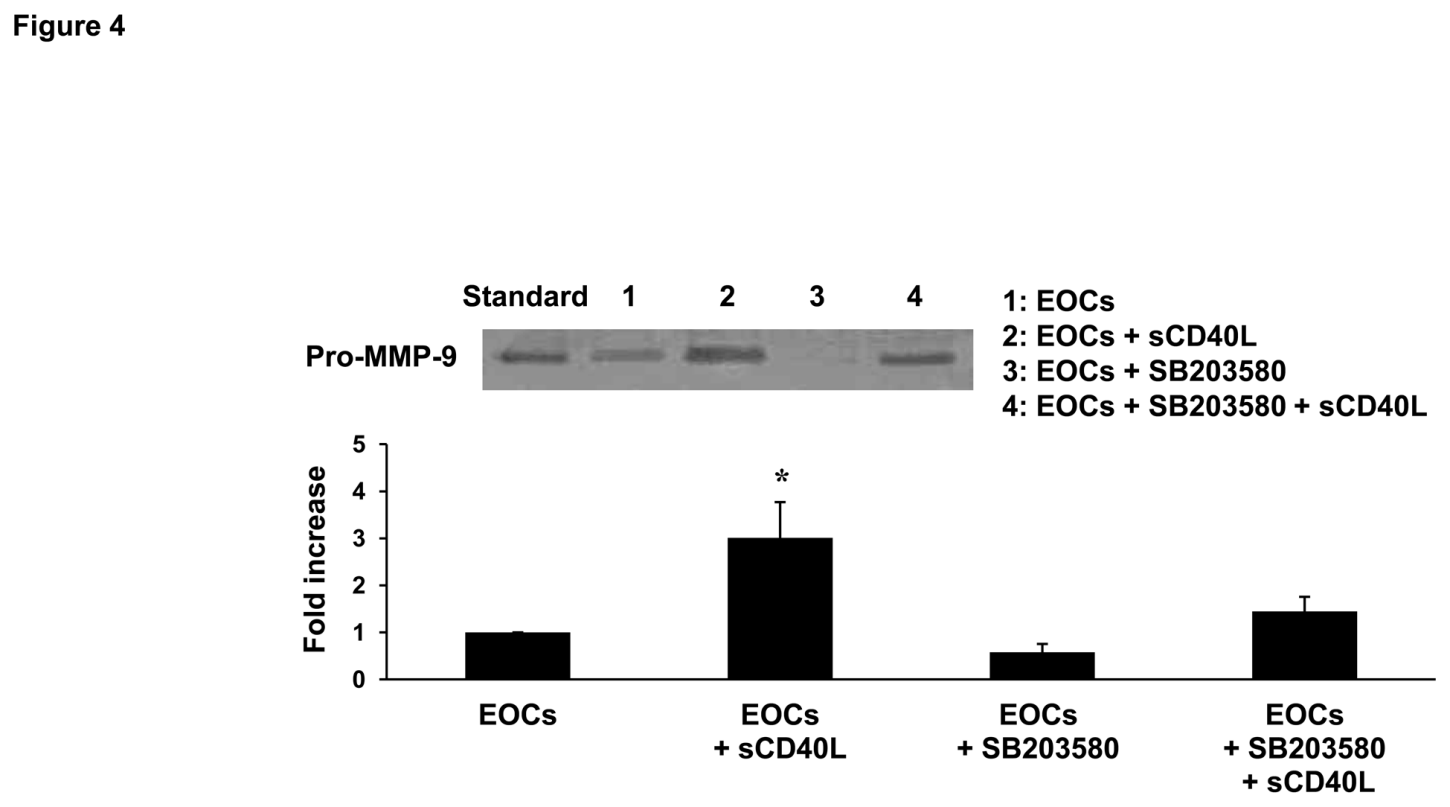
Enhancement of MMP-9 release in sCD40L-stimulated EOCs is p38 MAPK-dependent. Representative gelatin zymography gel of supernatants of EOCs and sCD40L (1 μg/mL)-, p38 inhibitor SB203580 (10 μM)-, and double sCD40L- and SB203580-treated EOCs. Histogram represents the mean of data ± SEM of fold increase in optical density over control EOCs. n ≥ 3, *P<0.05 *vs*. other groups.

 Having shown that sCD40L induces the release of MMP-9 and influences phosphorylation of p38 MAPK in EOCs, we were interested in investigating whether a correlation between these observations could be found. Indeed, using the specific p38 MAPK inhibitor, SB203580, we showed that inhibition of p38 MAPK phosphorylation significantly reversed the increase in MMP-9 release by EOCs in response to sCD40L (Figure 4).

### sCD40L enhances endothelial wound healing *via* increased MMP-9 release

**Figure 5:**
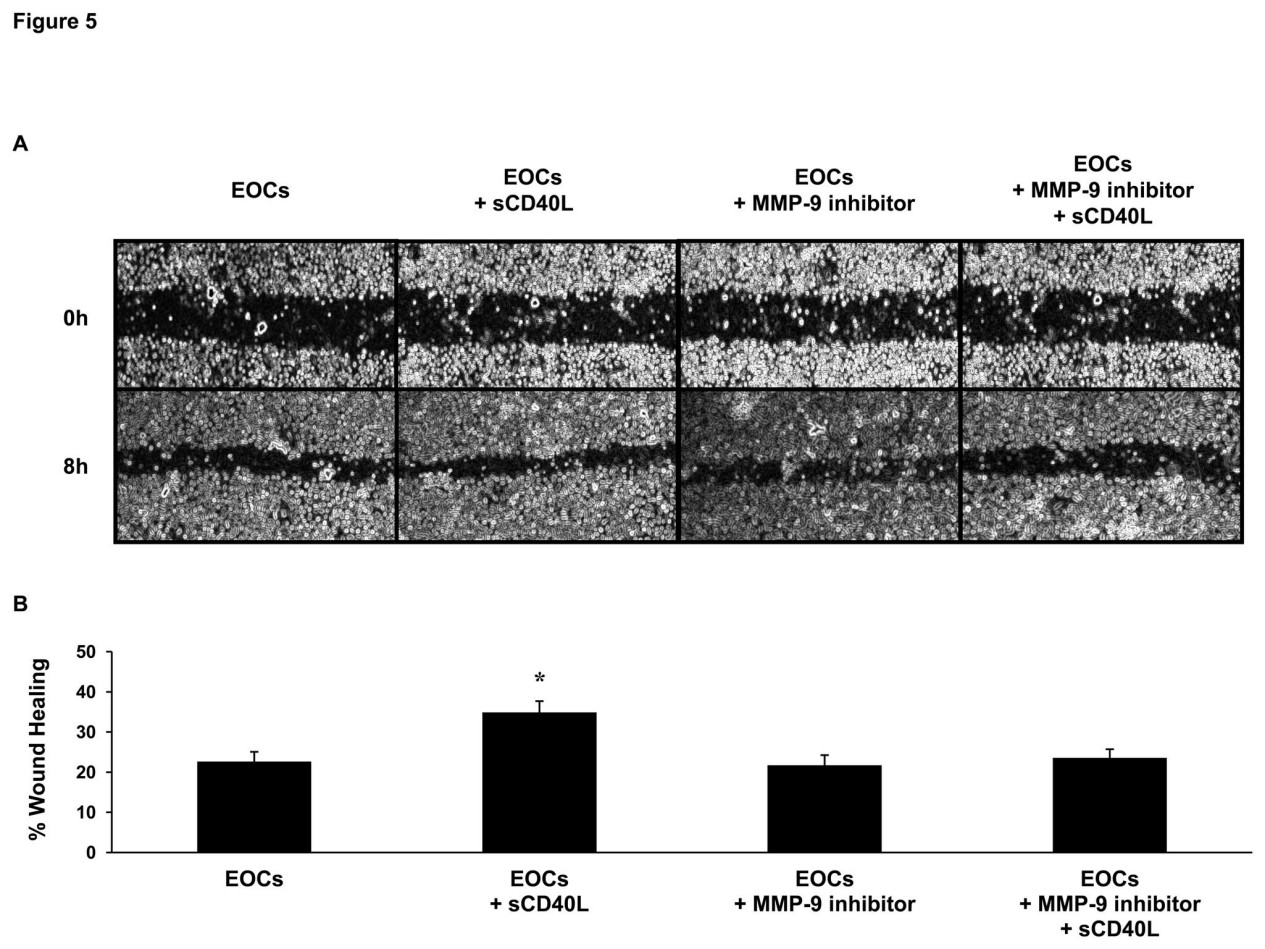
sCD40L-stimulated EOCs enhance endothelial wound healing *via* MMP-9 release. A) Representative images of HUVEC wound scratch assays at 0 and 8 hours in the presence of supernatants from EOCs and sCD40L (1 μg/mL)-, MMP-9 inhibitor (10 μM)-, and double sCD40L- and MMP-9 inhibitor-treated EOCs. B) Histogram represents the mean of data ± SEM of percent wound healing of HUVECs of overlay experiments. n ≥ 3, *P<0.05 *vs*. other groups.

 In order to further investigate the functional effects of sCD40L on EOCs, an *in vitro* wound scratch assay on HUVECs was performed with the supernatants from sCD40L-treated EOCs. Interestingly, supernatants from sCD40L-treated EOCs significantly favored endothelial wound healing *via* an increase in MMP-9 release by EOCs, as inhibition of MMP-9 activity reversed this effect (Figure 5). 

## Discussion

 Endothelial dysfunction or endothelial damage is a leading phenomenon in numerous cardiovascular diseases. Various studies have focused on developing approaches to regenerate the endothelium and induce novel vessel formation. The concept of neovascularization was first introduced by Asahara et al. [5]. This pioneer work showed that a rare population of CD34^+^VEGFR2^+^ cells, referred to as EPCs, circulate among adult human peripheral blood cells, have the capacity to differentiate into functionally mature endothelial cells *in vitro* and contribute to neoangiogenesis *in vivo*. Subsequent studies focusing on a subset of early EPCs or EOCs have suggested an indirect implication of these cells in endothelial repair and neovascularization through the release of paracrine angiogenic factors [12,35]. 

 We have previously shown that EPCs bind platelets and inhibit platelet adhesion, activation and aggregation, as well as thrombus formation [17]. In turn, platelets have been shown to promote recruitment and homing of EPCs to sites of vascular injury through the release of various mediators [36-38]. Among the mediators released by activated platelets, sCD40L, a thrombo-inflammatory mediator, has been identified as a potential biomarker of cardiovascular risk and disease. It has been shown that increased levels of sCD40L impair the function of EOCs by inducing an increase in superoxide anion production, thereby leading to reduced viability and proliferation [30]. In addition, we have previously shown that sCD40L reduces the anti-platelet properties of EOCs *via* release of free oxygen radicals [18]. In the present study, we shed light on the functional effects of sCD40L on EOCs and endothelial repair, which may have an important impact on patients with elevated levels of circulating sCD40L.

 The first finding of the present study demonstrates that EOCs express CD40 and its signaling adaptors molecules, the TRAF members. To our knowledge, the expression of TRAF members in EOCs and their association with CD40 following sCD40L stimulation had yet to be investigated. CD40 can bind five of the seven TRAF family members (TRAF1, 2, 3, 5, and 6), while TRAF4 and TRAF7 have not been reported to bind CD40. Here, we showed the presence of TRAF1, 2, and 3 in EOCs, whereas TRAF6 was expressed in PBMCs but lost during their differentiation into EOCs, which suggests that it may be involved in the initial steps of the differentiation process. The role of TRAFs in EOCs remains unexplored, but it is likely that sCD40L modulates EOC function by inducing association of different TRAF members to CD40. In this regard, it was pertinent to determine the effect of sCD40L on the expression of TRAFs and their association with CD40. We found that among the TRAF members present in EOCs, only TRAF1 expression was up-regulated and TRAF2 association with CD40 increased upon engagement of EOCs with sCD40L. TRAF1 weakly binds CD40, however this occurs indirectly *via* heterotrimerization with TRAF2 [39]. Studies on TRAF1 have often led to a variety of contradictory conclusions about whether it is a positive or negative regulator of TNF receptor signaling. These discrepancies may be due to the different cell types used in different studies. For instance, it is well established that TRAF1 regulates the binding of other TRAF members and facilitates downstream signaling, rather than inducing CD40 signaling by itself [39,40]. This may explain its up-regulation following sCD40L stimulation in EOCs. In both immune and endothelial cells, downstream CD40 signaling is triggered by the association of TRAFs to CD40 [23]. In our study, only TRAF2 association with CD40 increased in sCD40L-stimulated EOCs, suggesting that the CD40L/CD40/TRAF2 axis may trigger downstream signals, while TRAF1 may acts as a regulator of CD40 signaling [41]. The functional significance of CD40/TRAF signals in cells includes stimulation of kinases, gene expression, production of antibodies and a variety of cytokines, expression and up-regulation of adhesion molecules, and modulation of apoptosis. These various pathways can culminate in either induction or inhibition of biological functions. In the present study, we showed that sCD40L induces an activating signal involving at least p38 MAPK, as demonstrated by its phosphorylation in sCD40L-stimulated EOCs. Interestingly, we found that inhibition of p38 MAPK reverses the increased release of MMP-9 in sCD40L-stimulated EOCs, thus providing evidence that MMP-9 release by EOCs in response to sCD40L involves the p38 MAPK pathway. The same pathway has already been shown to be involved in platelet activation by sCD40L, which involves TRAF2/Rac1/p38 MAPK [22] and NF-kappaB [33]. However, the possibility that other signaling pathways may be involved in sCD40L-stimulated EOCs should not be excluded. In this regard, it has been shown that endothelial CD40, through activation of the PI3K/Akt signaling pathway, regulates cell survival, proliferation, migration, and vessel-like structure formation [42]. For instance, the link between TRAFs and p38 MAPK signaling in EOCs was not elucidated in the present study and further investigation will be required to establish such a connection.

 The process of neovascularization requires degradation of the extracellular matrix by matrix degrading enzymes, namely MMPs [14], which have also been shown to promote vascular repair [43]. We found that EOCs release MMP-9, which is further promoted by sCD40L stimulation. Based on various studies showing that EOCs are involved in angiogenesis and endothelial repair through the release of paracrine factors [12,35], we showed that sCD40L-treated EOCs promote endothelial wound repair *via* an increase in MMP-9 release. Hence, we correlated the functional role of EOCs in endothelial repair to their release of MMP-9 and identified p38 MAPK as an intracellular signaling mediator involved in the response of EOCs to sCD40L/CD40 stimulation. Thus, increased levels of sCD40L, released mainly by activated platelets, may induce the pro-angiogenic functions of EOCs on mature endothelial cells and contribute to endothelial repair. These findings are in contrast with a previous study demonstrating that sCD40L impairs EOC function *via* increased superoxide anion production and accelerates neointimal progression [30]. In addition, we have also shown that sCD40L impairs the anti-platelet properties of EOCs [18]. Thus, the negative impact of sCD40L on the modulation of platelet responses by EOCs and its positive impact on endothelial repair may be critical in coronary patients with elevated levels of circulating sCD40L. The net outcome in these patients may be related to an unbalance between these two functions: platelet activation and endothelial repair. Further studies are however required to validate this hypothesis and to further explore the effects of sCD40L on EOC function in *in vivo* settings involving animal models of atherothrombosis.

 In conclusion, we have investigated the effect of sCD40L on the function of EOCs in endothelial repair and demonstrated that the sCD40L/CD40/TRAF/p38 MAPK axis regulates MMP-9 release and positively impacts endothelial repair in EOCs. This study offers new insights into the pro-angiogenic role of EOCs in the presence of elevated levels of sCD40L in the circulation, as such occurring in patients with cardiovascular and atherothrombotic disease. 
